# Right ventricular shotgun pellet embolism: Case report and radiological aspect

**DOI:** 10.1016/j.radcr.2021.07.060

**Published:** 2021-08-25

**Authors:** Alain Abdo, Mylene Zamoun, Teodor Vasile, Minh Tam Bailly, Safwane El Hatimi, Marie-France Bellin, Olivier Meyrignac

**Affiliations:** a Radiology department, Bicetre Hospital APHP, 78 Rue du Général Leclerc, Le Kremlin Bicetre 94270, France; b Cardiology department, Bicetre Hospital APHP, Le Kremlin Bicetre, France; c Faculty of medicine, Paris-Saclay University, Le Kremlin Bicetre, France; d BioMaps Multimodal biomedical imaging laboratory, Paris-Saclay University, Le Kremlin Bicetre, France

**Keywords:** Computed tomography, Electrocardiogram gating, Penetrating injury, Bullet migration, Temporal resolution

## Abstract

Pellet embolism to the heart following gunshot injuries is an unusual event that requires a fast diagnosis. Imaging assessment is necessary to locate the projectiles and look for associated injuries. We present a case of a 41-year-old woman admitted after sustaining 2 gunshot wounds in the abdomen and left thigh, with the initial computed tomography (CT) scan showing a metallic object next to the right ventricle. Further radiological evaluation included transthoracic echocardiography and electrocardiogram-gated cardiac CT scan which confirmed the diagnosis of a migrating pellet to the right ventricle, entrapped within the trabeculations. Electrocardiogram-gated cardiac CT has a major role in detailed evaluation of bullet embolism to the heart cavities and guides the management.

## Introduction

The mechanism of gunshot related injuries is variable depending on the type of firearms and their projectile [1]. With each shot, rifles and handguns fire a single bullet whereas shotguns fire a cartridge of pellets which rapidly spread after leaving the barrel. The severity of the injury also depends on the distance between the firearm and the target. Intravascular missile migration is one of the serious complications that can be life threatening [2,3]. Radiological evaluation should not be delayed to assess the tissue damage and to detect such complications. Here we report a case of an intravascular pellet embolism to the right ventricle following shotgun injury diagnosed by electrocardiogram (ECG)-gated cardiac computed tomography (CT).

## Case report

A 41-year-old woman presented to the emergency room after sustaining 2 shotguns wounds in the abdomen and left thigh from a short distance. Upon admission, the patient was hemodynamically stable with a normal blood pressure and respiratory rate. Physical examination showed multiple ballistic wounds in the abdomen, both thighs and groins, associated with a deep wound on the upper-outer side of the left thigh. No exit wound was identified. Palpation found an upper abdominal guarding. Peripheral pulses were normally palpated in the 4 extremities.

**Fig. 1:**
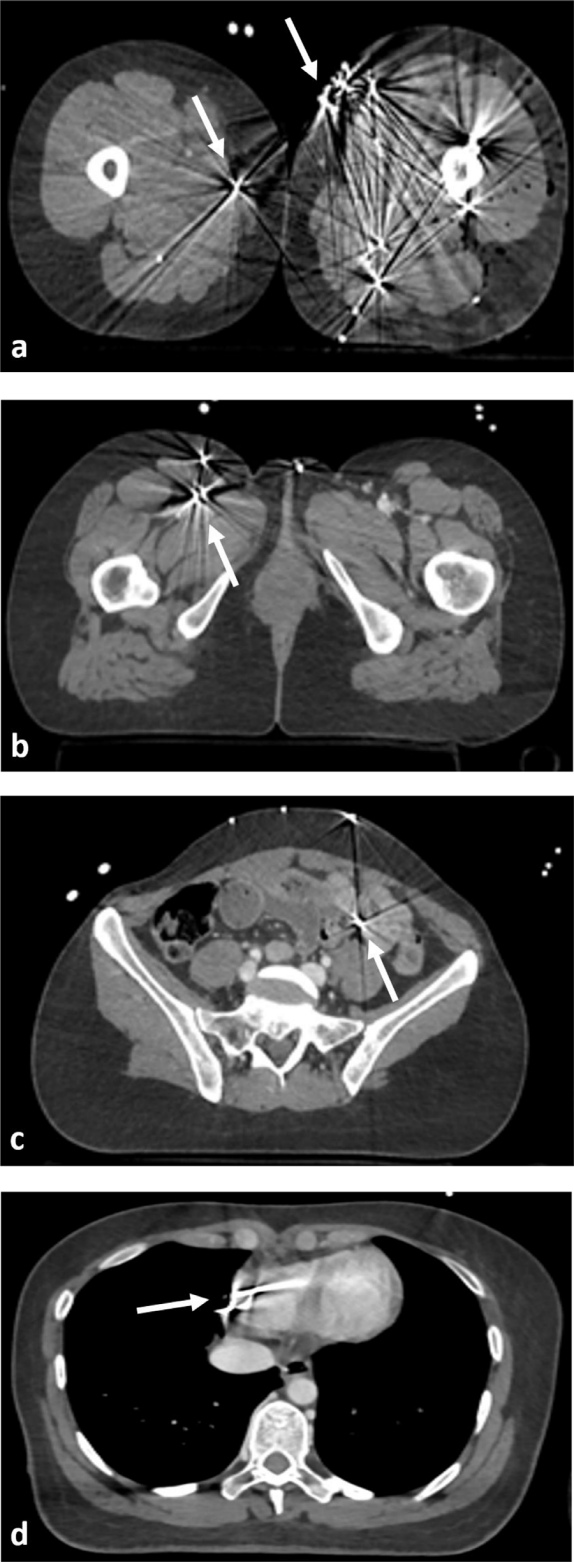
Axial CT slices showing multiple pellets (white arrows) within both thighs (A), in the right groin adjacent to femoral vessels (B) and inside the peritoneal cavity (C). At the level of the chest (D), axial slice showing metallic foreign body (white arrow) at the right ventricular wall for which exact location could not be assessed due to metallic and cardiac motion artifacts.

No abnormality was detected on the focused assessment with sonography for trauma. Whole-body CT scan using spiral acquisition with intravenous contrast administration and iterative algorithm for metal artifact reduction reconstruction was performed. It showed multiple small pellets within thighs, groins, abdominal wall and inside the abdomen and pelvis (Fig. 1). Mild pneumoperitoneum was present without any evident associated organ or vascular injuries. At the supra-diaphragmatic level, an isolated metallic foreign body was seen at the lower part of the right ventricular wall (Fig. 1). Its exact location was unknown due to metallic artifacts and cardiac motion, the patient was tachycardiac with a heart rate of 115 during the acquisition.

**Fig. 2:**
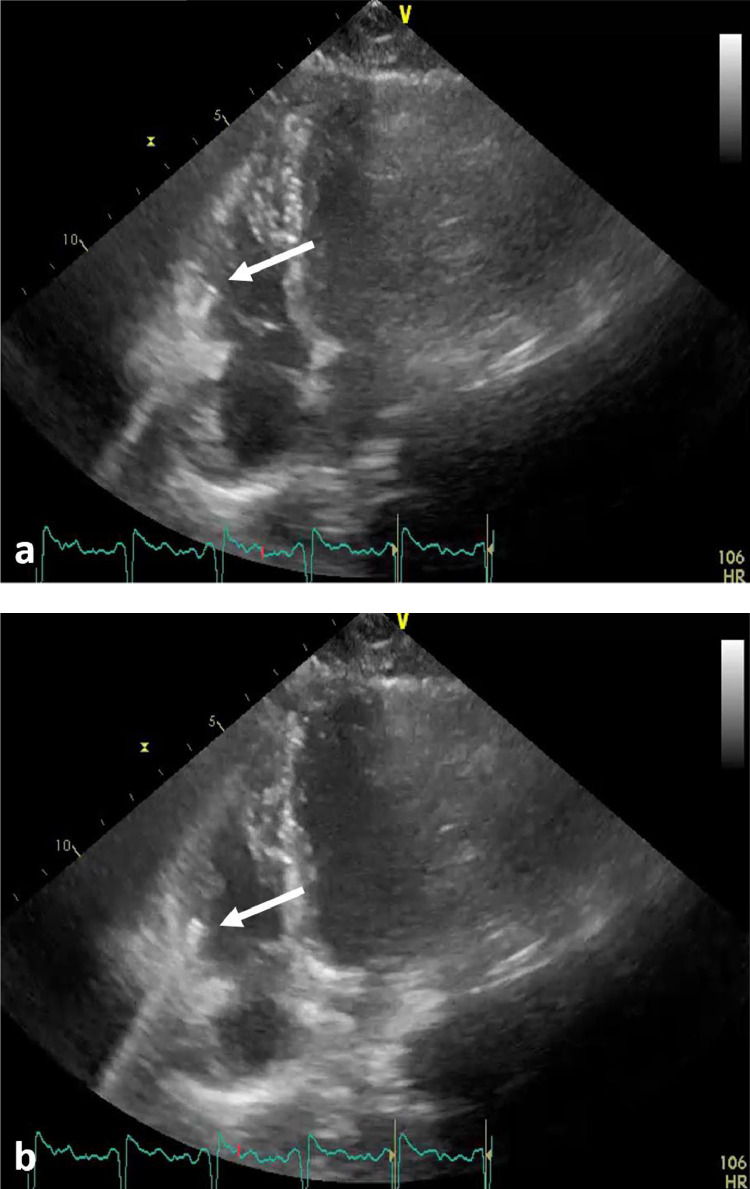
TTE with 4 chambers view showing a small hyperechogenic foreign body (white arrows) within the right ventricular wall causing posterior acoustic shadowing at 2 different phases of the cardiac cycle (A and B).

The patient underwent a laparotomy which showed no evidence of digestive or vascular injury. The transthoracic echocardiogram (TTE) showed a small echogenic foreign body, located in the infero-lateral wall of the right ventricle, causing posterior acoustic shadowing (Fig. 2). No pericardial effusion was detected. The patient was hospitalized for the next 2 weeks for wound debridement and care.

The metallic object seen on CT scan and TTE could be a penetrating pellet which directly reached the mediastinum. Another possibility is a migrating pellet through the venous system to the right heart cavities even though there were no evident associated vascular injuries seen on the whole-body CT scan.

**Fig. 3:**
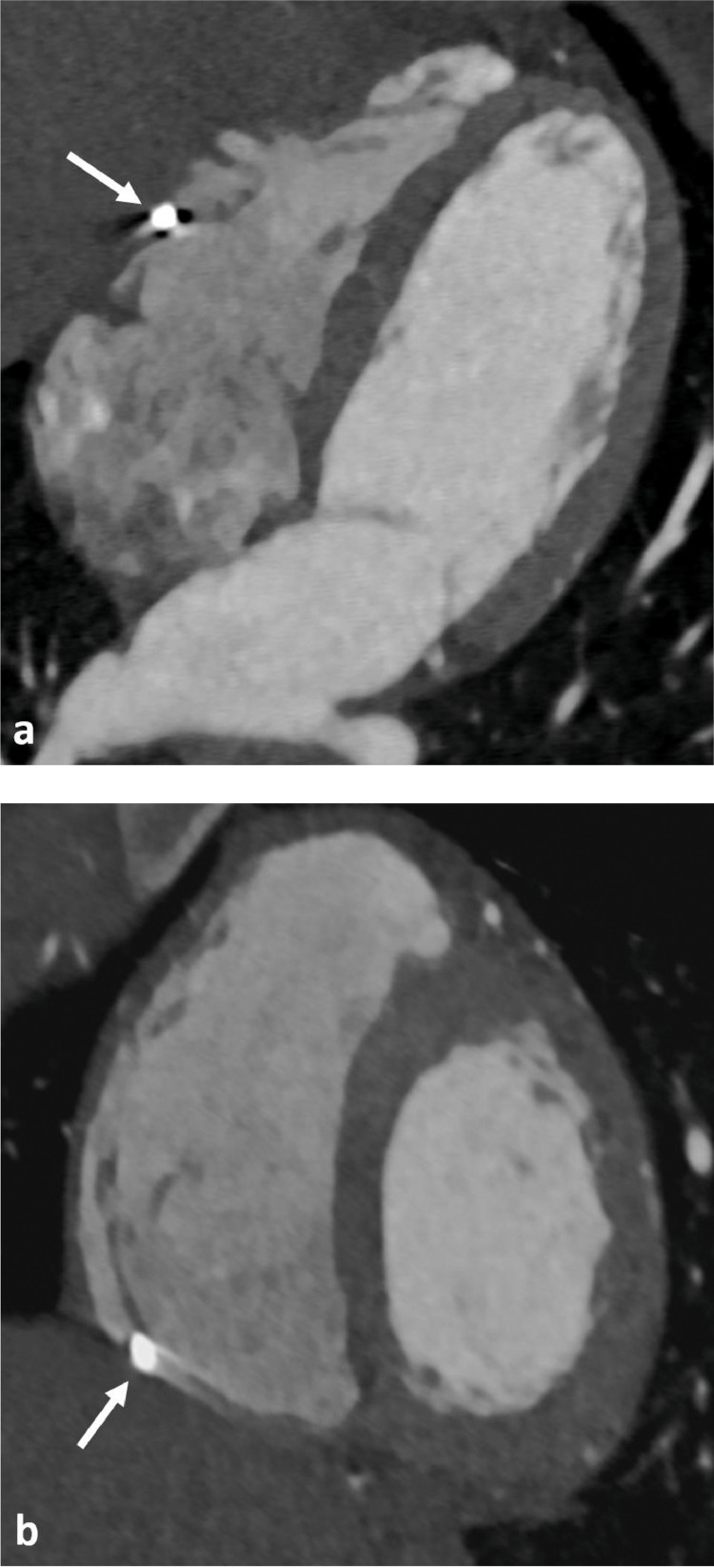
ECG-gated Cardiac CT: 4 chambers view (A) and short axis view (B) showing a small pellet (white arrows) completely intracavitary entrapped within the right ventricular trabeculations. The metallic artifacts and cardiac motion are significantly reduced with ECG-gating during the acquisition.

ECG-gated Cardiac CT scan was performed 5 days after admission using retrospective gating and iterative algorithm for metal artifact reduction reconstruction after administrating 10 mg of intravenous Tenormine (Atenolol) reducing the heart rate from 100 to 75. It confirmed the presence of a right ventricular intracavitary metallic pellet measuring 3 mm entrapped within the trabeculations throughout the cardiac cycle (Fig. 3). There was no evidence of a penetrating thoracic injury or pericardial abnormality. The most likely diagnosis was therefore a metallic pellet embolism to the right ventricle migrated from the femoral veins.

TTE was repeated 3 days later during the hospitalization showing the pellet within the same location in the right ventricle. It was decided after a multidisciplinary team meeting to follow a conservative management and to start prophylactic anticoagulation for 1 month. It was controlled by serial TTE rather than CT scan to avoid radiation exposure, looking for signs of stability, migration or myo-pericardial complications. On the first 2 monthly follow-ups, it was stable and no further investigations or workups were done.

## Discussion

Shotgun injuries can be severe at a short range. The pellets with high energy spread over a small area causing devastating damage [1]. Imaging assessment is necessary to locate the projectiles, to identify their pathway and to assess tissue and organ damage [2,3]. Initial radiological work-up should include conventional radiography with 2 perpendicular views. CT scan is often performed for preoperative planning and it can provide scout views as an alternative to conventional radiography. Additional radiological studies can be needed depending on the findings.

Intravascular pellet embolism is an unusual complication. It can be suspected whenever radiologic investigations show the pellets in an unexpected location deviated from its predicted path with no exit wound [4,5]. Associated vascular injuries are inconstantly seen[5]. The embolism is often anterograde following the direction of blood flow. Arterial emboli are symptomatic in 80% of cases and can result in ischemia, thus requiring urgent diagnosis [6]. Most of venous emboli migrate to the pulmonary arteries and are often asymptomatic and discovered incidentally. Lodgment in the right heart cavities is unusual [5,7,8].

The diagnosis of cardiac pellet embolism is often suspected on Chest x-ray then confirmed by TTE or transesophageal echocardiogram. Fluoroscopy can differentiate pericardial from intra-cavitary projectiles by their motion [5,8,9]. CT scan of the chest is helpful to assess the trajectory of the missile, evaluate vascular structures and assess lung parenchyma, but is not adequate to analyse bullet close to the heart because of metallic artifacts and cardiac motion [9].

In our case, both CTs were performed using a 3rd generation high pitch dual energy multi-detector CT scanner (Somatom Force, Siemens Medical Systems, Forchheim, Germany). One study showed that high pitch routine chest CT scan using such machines provides high temporal resolution allowing high quality visualization of cardiovascular structures and significantly reduces motion artifacts without the need of ECG synchronization [10]. However, in the first exam, cardiac structures analysis was limited due to tachycardia thus the need of another acquisition using ECG-gating and reducing the heart rate.

To the best of our knowledge, ECG-gated cardiac CT scan was not used for the diagnosis of bullets migration to the heart in the previously published case reports. This case showed that it was superior to the TTE and routine chest CT in evaluating the exact size of the pellet and assess its relation to heart chambers by significantly reducing artifacts.

Concerning the management of cardiac bullet emboli, no official consensus or guidelines exist [8]. The following recommendations are proposed from collating the previous publications. Missiles in the left ventricle should always be removed unless asymptomatic, small in size and completely embedded within the ventricular wall [9]. Small emboli to the right heart (<5mm) can be treated conservatively even if completely intracavitary or partially embedded because their migration with result in distal pulmonary embolism with a very low risk of being life threatening [11,12]. Whereas for larger emboli, their removal is recommended either by endovascular approach or surgery to avoid complications like massive pulmonary embolism, venous obstruction, arrhythmias, valvular dysfunction or myocardial irritability [8,9,12].

In our case, the pellet was less than 5 mm in size and completely intacavitary entrapped within the trabeculations of the right ventricle. Thus, a conservative management was chosen. It is to note that MRI for this patient is contraindicated because of the associated risk of projectile effect, twisting and burning [13].

## Conclusion

Gunshot pellet embolism to the heart is an unusual event that requires a fast diagnosis and adequate radiological investigations. ECG-gated cardiac CT is important to confirm the diagnosis and to assess the relation of the bullet to the heart structures. The decision to remove the pellets or not depends on the clinical situation, the size of the emboli and their location. In our case, the pellet was small in size and stable, which favored the decision of conservative management.
